# Influence of CYP2D6 phenotype on adherence, adverse effects, and attitudes in aripiprazole and risperidone users

**DOI:** 10.1017/neu.2025.11

**Published:** 2025-03-25

**Authors:** Elina Hietala, Anssi Solismaa, Markku Lähteenvuo, Ari V. Ahola-Olli, Katja Häkkinen, Kimmo Suokas, Erkki Isometsä, Jaana Suvisaari, Tuula Kieseppä, Minna Holm, Jari Tiihonen, Jouko Lönnqvist, Jarmo Hietala, Asko Wegelius, Kaisla Lahdensuo, Willehard Haaki, study SUPER-Finland, Olli Kampman

**Affiliations:** 1 Faculty of Medicine and Health Technology, Tampere University, Tampere, Finland; 2 Vanha Vaasa Hospital, Department of Forensic Psychiatry, Vaasa, Finland; 3 The Wellbeing Services County of Ostrobothnia, Department of Psychiatry, Seinäjoki, Finland; 4 Department of Psychiatry, Pirkanmaa Hospital District, Tampere, Finland; 5 Department of Forensic Psychiatry, University of Eastern Finland, Niuvanniemi Hospital, Kuopio, Finland; 6 Institute for Molecular Medicine Finland (FIMM), HiLIFE, University of Helsinki, Helsinki, Finland; 7 Faculty of Social Sciences, Tampere University, Tampere, Finland; 8 Department of Psychiatry, University of Helsinki and Helsinki University Hospital, Helsinki, Finland; 9 Mental Health Team, Finnish Institute for Health and Welfare, Helsinki, Finland; 10 Hospital District of Helsinki and Uusimaa, Helsinki, Finland; 11 Department of Clinical Neuroscience, Karolinska Institutet, and Center for Psychiatry Research, Stockholm City Council, Stockholm, Sweden; 12 Finnish Institute for Health and Welfare (THL), Helsinki, Finland; 13 University of Helsinki, Helsinki, Finland; 14 University of Turku, Faculty of Medicine, Department of Clinical Medicine (Psychiatry), Turku, Finland; 15 Department of Psychiatry, Turku University Hospital, Turku, Finland; 16 Mehiläinen, Helsinki, Finland; 17 Molecular Medicine and Surgery, Karolinska Institutet, Stockholm, Sweden; 18 Department of Psychology and Logopedics, Faculty of Medicine, University of Helsinki, Helsinki, Finland; 19 Department of Psychiatry, Research Unit of Clinical Neuroscience, University of Oulu, Oulu, Finland; 20 Department of Psychiatry, Oulu University Hospital, Oulu, Finland; 21 Neuroscience Center, HiLIFE, University of Helsinki, Helsinki, Finland; 22 Department of Clinical Sciences, PsychiatryUmeå University, Umeå, Sweden; 23 Massachusetts General Hospital Massachusetts General Hospital, Boston, MA, USA; 24 Broad Institute of MIT and Harvard, Cambridge, MA, USA

**Keywords:** Psychotic disorders, treatment adherence and compliance, cytochrome P-450 CYP2D6, pharmacogenetics, antipsychotic agents

## Abstract

Non-adherence and negative attitudes towards medication are major problems in treating psychotic disorders. Cytochrome P450 2D6 (CYP2D6) contributes to the metabolism of aripiprazole and risperidone, and variations in CYP2D6 activity may affect treatment response or adverse effects. However, the impact of these variations on adherence and medication attitudes is unclear. This study investigates the relationships between CYP2D6 phenotype, self-reported adherence, adverse effects, and attitudes among aripiprazole and risperidone users. The study analysed data from the SUPER-Finland cohort of 10,474 adults with psychotic episodes, including 1,429 aripiprazole and 828 risperidone users. The Attitudes towards Neuroleptic Treatment (ANT) questionnaire assessed adherence and adverse effects in all patients, while medication-related attitudes were examined in a subgroup of 1,000 participants. Associations between CYP2D6 phenotypes and outcomes were analysed using logistic regression and beta regression in aripiprazole and risperidone groups separately. Among risperidone users, we observed no association between CYP2D6 phenotypes and adherence, adverse effects, or attitudes. Similarly, we found no link between adherence and CYP2D6 phenotypes among aripiprazole users. However, aripiprazole users with the ultrarapid CYP2D6 phenotype had more adverse effects (OR = 1.71, 95 % CI 1.03–2.90, *p* = 0.041). Among aripiprazole users, CYP2D6 ultrarapid phenotype was associated with less favourable attitudes towards antipsychotic treatment (β = −0.48, *p* = 0.023). These findings provide preliminary evidence that the ultrarapid CYP2D6 phenotype is associated with increased adverse effects and negative attitudes towards antipsychotic medication among aripiprazole users. CYP2D6 phenotype did not influence adherence, adverse effects, or attitudes among risperidone users.

## Graphical abstract

**Figure:**
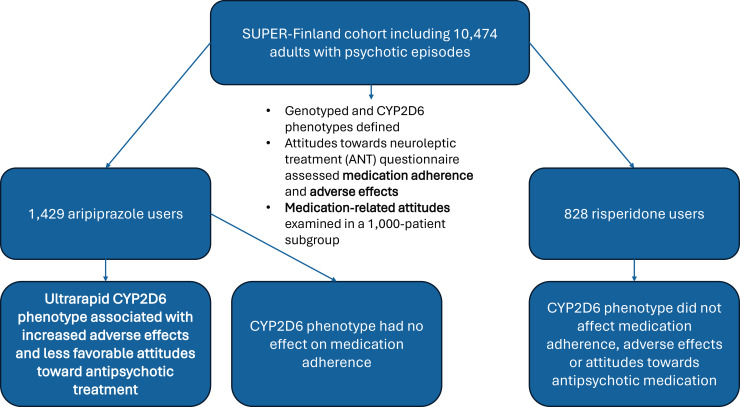

Significant outcomes

- We found preliminary evidence that the ultrarapid CYP2D6 phenotype is associated with adverse effects and negative attitudes towards antipsychotic medication among patients using aripiprazole.
- No associations between CYP2D6 phenotype and self-reported adherence, adverse effects and attitudes were found among risperidone users.

Limitations

- The ANT scale was available only for a subgroup (n = 1000) of patients, which limits the generalizability of the findings regarding medication-related attitudes.
- This study was cross-sectional and did not include data on previous antipsychotic treatments or baseline measurements for the studied outcomes. The sample included more long-term patients and lacked data on current psychopathology, which may affect the interpretation of results.
- The study relies on self-reported data, which may introduce bias and affect the reliability of the results.

## Introduction

Psychotic disorders are major public health concerns worldwide (Charlson *et al*., 2018; Same *et al*., 2023). Antipsychotic medication is a core of the successful treatment for these disorders (Jauhar, *et al*., 2022). Discontinuation of antipsychotic medication increases the risks of psychosis relapse, rehospitalisation, and death, whereas long-term antipsychotic use is associated with increased survival (Tiihonen, *et al*., 2018). Despite this, early discontinuation is common: 40 % of first-episode schizophrenia patients discontinue their medication immediately after hospitalisation, and over 50 % within two months of discharge (Tiihonen *et al*., 2011)

Detecting and predicting medication non-adherence is challenging due to its complex nature (Leijala *et al*., 2021). Several factors like previous non-compliance, substance abuse, and adverse effects contribute to non-adherence (Fenton *et al*., 1997; De Millas *et al*., 2006; Haddad *et al*., 2014; Nyanyiwa *et al*., 2022). Negative attitudes towards antipsychotic medication have a moderate to strong association with non-compliance. Previous problems with medication adherence predict future adherence issues. (Fenton, *et al*., 1997) Negative attitudes towards antipsychotics can lead to treatment discontinuation, potentially affecting treatment outcomes (Kampman *et al*., 2002; Leijala *et al*., 2021; Townsend *et al*., 2022).

Antipsychotic medications vary in their tolerability (Leucht *et al*., 2013) with large interindividual variability, and differences in adverse effects between antipsychotic medications are more profound than efficacy differences (Huhn *et al*., 2019). Poor tolerability may result in multiple failed treatment attempts before finding a favourable balance between symptom management and adverse effects (Pouget *et al*., 2014). A positive experience of using medication stemming from positive therapeutic effects and low incidence of adverse effects is likely to have a positive impact on attitudes towards antipsychotic medication (Freudenreich *et al*., 2004; Lambert *et al*., 2004).

Genetic factors, especially differences in cytochrome P450 (CYP) enzymes, largely explain the variability in antipsychotic-related adverse effects (Spear *et al*., 2001; Murray, 2010; Pouget *et al*., 2014; Caudle *et al*., 2020). CYP2D6 is one of main enzymes in the metabolism of several antipsychotics (e.g., aripiprazole, iloperidone, paliperidone, risperidone, chlorpromazine, fluphenazine, haloperidol, loxapine, perphenazine, thioridazine, and zuclopenthixol), and makes a contribution to the metabolism of olanzapine and clozapine (Ravyn *et al*., 2013). Variability in CYP2D6 function is common and mainly caused by genetic differences like polymorhisms, gene copy number variations, and structural rearrangerments. To measure the extent of how CYP2D6 polymorphisms affect metabolism, individuals can be phenotyped as poor (PM), intermediate (IM), normal (NM), and ultra-rapid CYP2D6 metabolizers (UM) (Caudle *et al*., 2020). UMs may have difficulties in reaching therapeutic drug serum levels while PMs may be prone to excessive levels and increased adverse effects (Jukic *et al*., 2019; Beunk *et al*., 2023). Similarly, using CYP2D6 inhibitors may elavate drug concentrations (Lisbeth *et al*., 2016).

Aripiprazole and risperidone are widely prescribed antipsychotic medications (Pringsheim & Gardner, 2014; Højlund *et al*., 2019; Finnish Medicines Agency Fimea and Social Insurance Institution, 2021), primarily metabolised by CYP2D6 and partially by CYP3A4 (Hiemke *et al*., 2018). These medications are also commonly utilised for non-psychotic indications, such as mood disorders and behavioural disturbances (Højlund *et al*., 2019). Variances in CYP2D6 genotype and concomitant use of CYP2D6 inhibitors, along with CYP3A4 inducers or inhibitors, can result in diverse metabolism and exposure levels of aripiprazole and risperidone (Jukic *et al*., 2019).

Patients with poor or ultrarapid CYP2D6 phenotype, or those using CYP2D6 inhibitors, have reported difficulties in medication tolerability and efficacy (Jukic *et al*., 2019), potentially affecting adherence and attitudes towards antipsychotic medication. However, contrasting this, a recent meta-analysis found limited associations between CYP2D6 variations and the adverse effects of aripiprazole and risperidone, suggesting that other factors may play a more significant role in influencing these outcomes (de Brabander *et al*., 2024).

Despite the importance of medication tolerability and efficacy on adherence, there is still a lack of evidence regarding the effect of pharmacogenetic phenotype on adherence, adverse effects, and medication-related attitudes (Jukic *et al*., 2019). This study aims to examine the influence of CYP2D6 phenotype on self-reported adherence, adverse effects, and medication-related attitudes among aripiprazole and risperidone users, while accounting for CYP2D6 inhibitor use. We anticipated that these outcomes would be affected by increased adverse effects in PMs and diminished treatment efficacy in UMs.

## Material and methods

### Participants

The study conducted using the data from the SUPER-Finland (Finnish study for the hereditary mechanisms behind psychotic illnesses, superfinland.fi) cohort (Lähteenvuo *et al*., 2023), a part of the Stanley Global Neuropsychiatric Initiative. The SUPER-Finland cohort was recruited during 2016-2018 and consists of 10,474 adult participants from Finland with a history of at least one psychotic episode. The participants were recruited throughout Finland from in- and outpatient psychiatric care, primary care, and housing units with a diagnosis of schizophrenia (International Classification of Diseases, 10^th^ revision (ICD-10) code F20), schizoaffective disorder (F25), bipolar disorder (F30, F31), major depressive disorder with psychotic features (F32.3 and F33.3), or other non-affective psychoses (Ahti *et al*., 2022). The study excluded minors and individuals unable to provide informed consent. Participants provided written informed consent. SUPER-Finland study cohort has been more comprehensibly documented in an independent article (Lähteenvuo *et al*., 2023).

### Study variables

#### Diagnosis of psychosis

In addition to self-reported diagnosis, the lifetime diagnoses of the study subjects were studied from Finnish Care Register for Health Care maintained by the Finnish National Institute for Health and Welfare. The register contains data from The Finnish Hospital Discharge register (1969-1993), followed by the Care Register for Health Care and The Register for Primary Health Care Visits. The register covers all inpatient hospital treatments in Finland from the year 1969, all specialised outpatient care visits from the year 1994, and since 2011 all outpatient primary health care delivered in Finland. The register displays good accuracy of mental health diagnoses (Sund, 2012).

Patients with schizophrenia spectrum disorders tend to receive other non-affective psychosis diagnoses in the beginning of their disease course (Maurer & Häfner, 1995; Kiviniemi *et al*., 2010). Thus, the diagnoses evolve towards more specific diagnostic titles over time. In this study a group of expert senior psychiatrists decided upon a hierarchical diagnosis algorithm to assign each patient a main diagnosis. The algorithm for diagnostic hierarchy prioritised the diagnosis in the following order: 1) schizophrenia (ICD-10: F20), 2) schizoaffective disorder (ICD-10: F25), 3) bipolar disorder (ICD-10: F30-31), 4) psychotic depression (F32.3, F33.3), 5) other nonaffective psychotic disorder (ICD-10: F22-F24, F28-F29) and 6) other mental disorders (ICD-10: F-codes not previously mentioned). The single hierarchically highest diagnosis that occurs at least once is considered the assigned main diagnosis. We calculated the years since the hierarchical diagnosis was first set to the study visit date for each patient.

#### Attitudes towards neuroleptic treatment (ANT) scale

We measured participants’ medication adherence, adverse effects, and attitudes using the ANT questionnaire, which is theoretically based on the dichotomous ‘Drug Attitude 10’ (DAI-10) questionnaire (Kampman *et al*., 2000). ANT-scale uses 12 Visual Analogue Scale (VAS) items (scores 0 to 100, higher scores reflect better attitudes). These scales consist of several statements regarding subjective positive and negative feelings about medication, adherence, adverse effects, insight, and attitudes towards antipsychotics.

The shorter ANT-S questionnaire is validated and includes eight items: five on drug attitudes, one on insight, one on adverse effects, and one on medication adherence (Leijala *et al*., 2021). In the SUPER-Finland cohort, a subgroup of 1,000 unselected patients from the Tampere region, one of the five university hospital regions in Finland, completed the ANT-S questionnaire. Out of these, 931 patients had CYP2D6 phenotype available.

Adherence was assessed with a question ‘Estimate how much of your prescribed psychiatric medication you have used in the last 4 weeks’, rated on a five-point scale from 0 % to 100 % with options ‘not at all’ (0 %), ‘a little’ (25 %), ‘half’ (50 %), ‘most of the medication’ (75 %) and ‘all’ (100 %). Responses were dichotomised into two groups: 100 % adherence and those who reported using less than all prescribed medication.

Medication adverse effects were assessed with a question of ‘Estimate how much your current medication causes adverse effects’, rated on a scale from ‘no adverse effects at all’ to ‘really a lot of troublesome adverse effects’, scored 0 to 10 (a higher score reflecting more adverse effects). This variable was dichotomised using the median of the linear adverse effects variable as a cutoff point: from ‘not at all’ to ‘a little’ adverse effects (scale 0-3), and from ‘quite a bit’ to ‘really a lot of troublesome’ adverse effects (scale 4-10).

We dichotomised the mean score across five ANT attitude items (ANT-attitude): importance of medication, willingness of taking medication, expected effect on present state, on thinking ability, and on autonomy (Kampman *et al*., 2000). Patients were divided into two groups based on this dichotomy: those scoring 58 or lower, representing the lower 25th percentile, and those scoring above 58, to focus on and examine the more negative attitudes.

#### Use of medication in defined daily dose

We collected information about the medications used by the participants through study interviews. We complemented self-reported medication information from the interviews with data from medical records and prescriptions whenever available. The collected medication data was converted to defined daily dose (DDD) ratios. DDD is an assumed average maintenance dose per day for a drug used for its main indication in adults. DDDs are produced by the WHO Collaborating Centre for Drug Statistics Methodology in Oslo, Norway (www.whocc.no/). DDDs for the studied antipsychotics are 15 mg for aripiprazole and 5 mg for risperidone. (WHO, 2022)

We calculated the DDD ratio using the DDD method, dividing the self-reported medication by the DDD (Leucht *et al*., 2016). For patients using multiple antipsychotics, we calculated the total DDD ratio by summing the DDD ratios for each antipsychotic agent.

In addition, for each patient, we calculated the total number of psychotropic medications, including antipsychotics, antidepressants, mood stabilisers, anxiolytics, and hypnotics.

#### Genotyping and defining the CYP2D6 phenotypes

The SUPER-Finland study participants have been genotyped with Illumina Global Screening Array (*n* = 10,075) containing 688,032 probes at Broad Institute in Cambridge, Massachusetts, USA. Participants with genotyping success rate<90 % and discordant reported gender and genotyped sex were excluded. Subsequently, variants with over 10 % of missing genotype calls were excluded. Pi-hat cut-off of 0.15 was used to exclude related samples. Variants deviating from Hardy-Weinberg equilibrium were excluded with p-value cut-off of *p*<1 × 10^−8^. Samples with low or excess heterozygosity (± 3SD) were excluded. Variants with minor allele frequency below 0.0001 were excluded. After these quality control steps, two rounds of imputation were performed. On the first round, we used population specific imputation panel to impute genetic variants. Data was pre-phased with Eagle 2.4 and then imputed with Beagle 4.1 software (Browning and Browning, 2016; Loh *et al*., 2016). To exclude poorly imputed variants, we filtered the imputed data set with INFO cut-off of 0.7. As *CYP2D6* copy-number is needed to estimate CYP2D6 phenotype from genotype, we performed a second imputation round by using Finnish BrePainGen sample as an imputation panel (n = 902). BrePainGen has been genotyped with Human Omni Express chip. *CYP2D6* copy-number variation (CNV) has been genotyped with real-time PCR (Cajanus *et al*., 2018) from BrePainGen participants. After quality control measures, we incorporated CNV information to Plink format GWAS data derived from OmniExpress chip by representing the CNV as two biallelic markers indicating whether a subject carried deletion or not and whether a subject carried a duplication or not. The biallelic markers were given a genomic position that corresponded to the start of *CYP2D6* gene. Then this combined data was converted to VCF format and phased against same reference genome than the SUPER-Finland data set during the first imputation round. Subsequently, the resulting BrePainGen data was used to impute *CYP2D6* CNV to SUPER-Finland. The CNV imputation methodology has been validated earlier and due to higher frequency of CYP2D6 in Finland compared to most other European populations, the imputation works especially well for detecting CYP2D6 UMs with 94.5 % of individuals as correctly phenotyped. The corresponding percentages for CYP2D6 IMs vas 84.0 %, for PMs 89.5 %, and for NMs 76.7 % (Häkkinen *et al*., 2022). In the last step, the imputed CNV and single nucleotide polymorphism data were used to infer CYP2D6 metabolic phenotype from genotype by following The Clinical Pharmacogenetics Implementation Consortium guidelines and classified as poor, intermediate, normal, and ultrarapid (Crews *et al*., 2021). Plink 1.9, Plink 2.0 and Bcftools software were used during the data handling and quality control. The following CYP2D6 star alleles were considered in the phenotyping: *3, *4, *5, *6, *10, *20, *41, *59. Patients were not informed of their CYP2D6 phenotype.

#### Use of CYP2D6 inhibitors, CYP3A4 inducers and inhibitors

Concurrent use of CYP2D6 inhibitors, CYP3A4 inducers and inhibitors can affect aripiprazole and risperidone metabolism. Notably, there are no known CYP2D6 inducers, and thus no established list of CYP2D6 inducers exists (Flockhart, 2003). CYP2D6 phenoconversion was performed by adjusting activity scores based on the use of strong or moderate CYP2D6 inhibitor drugs as defined by the Food and Drug Administration (FDA) (U.S. Food & Drug Administration (FDA), 2023). In patients receiving a strong CYP2D6 inhibitor (bupropion, cobicistat, fluoxetine, fluvoxamine, paroxetine, quinidine, or terbinafine), the activity score was multiplied by zero. This resulted in a phenotype of ‘Poor’ for all these patients. For patients on a moderate inhibitor (abiraterone, amiodarone, cinacalcet, duloxetine, lorcaserin, mirabegron, rolapitant, or vemurafenib) the activity score was multiplied by 0.5. CYP2D6 phenotypes were reassigned based on the adjusted activity score. (Cicali *et al*., 2021; Crews *et al*., 2021) We grouped patients based on whether they were receiving a CYP3A4 inducer or inhibitor, counting those drugs classified as either strong or moderate. Apalutamide, carbamazepine, enzalutamide, ivosidenib, lumacaftor, ivacaftor, mitotane, phenytoin, and rifampicin were considered strong CYP3A4 inducers. Bosentan, cenobamate, dabrafenib, efavirenz, etravirine, lorlatinib, pexidartinib, phenobarbital, primidone, and sotorasib were considered moderate CYP3A4 inducers. Ceritinib, clarithromycin, cobicistat, elvitegravir, idelalisib, indinavir, itraconazole, ketoconazole, lopinavir, nefazodone, nelfinavir, paritaprevir, posaconazole, ritonavir, saquinavir, telithromycin, tipranavir, voriconazole, isavuconazole, and verapamil were considered strong CYP3A4 inhibitors. Aprepitant, ciprofloxacin, conivaptan, crizotinib, diltiazem, dronedarone, erythromycin, fluconazole, and imatinib were considered moderate CYP3A4 inhibitors (U.S. Food & Drug Administration (FDA), 2023).

### Statistical analyses

For two variable comparisons, we used Pearson correlation to study the relationship between age and time from hierarchical diagnosis, the Mann-Whitney *U* test for age and antipsychotic total DDD, and chi-squared tests for differences in distributions of aripiprazole and risperidone users among different CYP2D6 phenotype groups. Logistic regression models were applied to assess the impact of CYP2D6 phenotype on self-reported medication adherence and side effects analysing aripiprazole and risperidone groups separately. Covariables were chosen to adjust for confounding factors and to isolate the effects of CYP2D6 phenotype (ultrarapid, normal, intermediate, and poor, with normal as the reference). The covariables included age, gender, body mass index, current smoking status, psychosis diagnosis, number of psychotropic medications, and living in supported housing. Research suggests notable differences between affective and non-affective psychoses in various aspects such as premorbid adjustment, clinical presentation, treatment response, cognitive function, and structural brain alterations (Bora, *et al*., 2010; Knöchel *et al*., 2016; Torrent *et al*., 2018). Thus, to streamline our models and prevent oversaturation, we dichotomised diagnoses into non-affective and affective psychosis groups rather than analysing each diagnostic category separately.

The correlation between ANT-attitude scores and medication adverse effects was analysed using Pearson’s correlation. To examine variations in ANT-attitude scores across different CYP2D6 phenotype groups, we utilised the Kruskal-Wallis and Mann-Whitney U tests. For the ANT attitude outcome, we performed stratified regression models for aripiprazole and risperidone users separately. Due to limited sample sizes for patients with available ANT data (n = 147 aripiprazole users; *n* = 92 risperidone users), the number of covariates was reduced to CYP2D6 phenotypes, age, gender, and number of psychotropic medications. As the outcome variable was right-skewed and residuals were not normally distributed in a linear regression model, we applied beta regression, using the ANT score divided by 100 to scale values between 0 and 1. We performed statistical analyses with R version 4.1.1 (R Core Team (2021). R: A language and environment for statistical computing. R Foundation for Statistical Computing, Vienna, Austria. URL https://www.R-project.org/)

## Results

### Participant demographics

Of the total 10,474 study participants at the time of recruitment 9,232 had CYP2D6 phenotype available and from those 931 filled the ANT-attitude scale. Table 1 displays the characteristics of the sample. All individuals included in the analyses concerning predicted CYP2D6 phenotype were unrelated. The groups were fairly similar in characteristics and distribution. Due to the small number of patients using any CYP3A4 inducers or inhibitors, these variables were excluded from subsequent regression models. The years since the current hierarchical diagnosis was set correlated strongly with age, thus only age was used in the following regression models (Pearson *r* = 0.67, *p*<0.0001). Among patients aged 58 years and older, representing the highest 25th percentile, DDD ratios were significantly lower compared to those younger (1.40 ± 1.14 vs. 1.57 ± 1.16; Mann-Whitney *U* test, *p*<0.0001). Table 2 displays the DDD ratios of aripiprazole and risperidone in different CYP2D6 phenotype groups. No difference was observed in the distribution of aripiprazole users and risperidone users between CYP2D6 phenotypes (χ^2^, *p* = 0.51 and *p* = 0.78, respectively), including comparisons of ultrarapid metabolizer or poor metabolizer groups to other CYP2D6 phenotypes in both aripiprazole and risperidone user groups (χ^2^, *p* > 0.05).

**Table 1.** Covariables in all cytochrome P450 2D6 (CYP2D6) genotyped patients and in the subgroup who filled Attitudes towards Neuroleptic Treatment (ANT)-scale

	All patients (*n* = 9232) mean ± SD or *n* (%)	Patients with ANT-scale available (*n* = 931) mean ± SD or *n* (%)	Patients with aripiprazole (n = 1429) mean ± SD or n (%)	Patients with risperidone (n = 828) mean ± SD or n (%)
Age (years)	46.7 ± 14.8	45.1 ± 15.4	42.3 ± 13.7	49.5 ± 16.0
Adherence: using all (100%) prescribed medication	7236 (80.7 %)	703 (77.0 %)	1159 (82 %)	662 (80.0 %)
Patient rated drug adverse effects (scale 0-10)^a^	3.7 ± 2.7	3.7 ± 2.7	4.0 ± 2.5	3.4 ± 2.7
Patients who had from ‘quite a bit’ to ‘really a lot of troublesome’ adverse effects (scale 4-10)^a^	4943 (55.4 %)	500 (54.8 %)	839 (59.6 %)	414 (51.7 %)
ANT-attitude		69.6 ± 16.8	70.3 ± 17.0	69.2 ± 18.8
Antipsychotic total DDD ratio	1.5 ± 1.2	1.3 ± 1.1	2.1 ± 1.2	1.3 ± 1.2
Risperidone DDD ratio	0.6 ± 0.4	0.6 ± 0.3	0.7 ± 0.4	0.6 ± 0.4
Aripiprazole DDD ratio	1.3 ± 0.6	1.2 ± 0.5	1.3 ± 0.6	1.4 ± 0.5
Number of psychotropic medications in use	2.4 ± 1.5	2.4 ± 1.5	3.1 ± 1.4	2.9 ± 1.4
Years since current hierarchical diagnosis was first set	17.6 ± 12.5	16.2 ± 12.5	14.9 ± 11.0	17.4 ± 14.0
Gender female	4581 (49.6 %)	473 (50.8 %)	770 (53.9 %)	391 (47.2 %)
Gender male	4651 (50.4 %)	458 (49.2 %)	659 (46.1 %)	437 (52.8 %)
BMI (kg/m^2^)	30.3 ± 7.0	30.4 ± 7.4	31.8 ± 7.9	30.0 ± 6.8
Diagnosis: Schizophrenia	5117 (55.4 %)	445 (47.8 %)	795 (55.6 %)	447 (54.0 %)
Diagnosis: Schizoaffective disorder	837 (9.1 %)	79 (8.5 %)	165 (11.5 %)	60 (7.2 %)
Diagnosis: Bipolar disorder	1417 (15.3 %)	146 (15.7 %)	213 (14.9 %)	74 (8.9 %)
Diagnosis: Other psychosis	932 (10.1 %)	117 (12.6 %)	145 (10.1 %)	165 (20.0 %)
Diagnosis: Psychotic depression	475 (5.1 %)	69 (7.4 %)	83 (5.8 %)	54 (6.5 %)
Diagnosis: Other mental disorder	454 (4.9 %)	75 (8.1 %)	28 (2.0 %)	28 (3.3 %)
Smoker currently^b^	3853 (44.2 %)	421 (45.6 %)	571 (40.0 %)	325 (39.2 %)
Strong CYP2D6 inhibitor in use	540 (5.8 %)	56 (6.0 %)	112 (7.8 %)	37 (4.4 %)
Moderate CYP2D6 inhibitor in use	202 (2.2 %)	25 (2.7 %)	38 (2.7 %)	14 (1.7 %)
One or multiple CYP3A4 inducer(s) in use	68 (0.67 %)	3 (0.32 %)	5 (0.35 %)	8 (0.1 %)
One or multiple CYP3A4 inhibitor(s) in use	13 (0.14 %)	1 (0.11 %)	1 (0.07 %)	3 (0.36 %)
Living in supported housing	2280 (24.7 %)	210 (22.6 %)	289 (20.2 %)	244 (29.5 %)
Aripiprazole in use	1429 (15.5 %)	147 (15.8 %)	1429 (100 %)	45 (5.4 %)
Risperidone in use	828 (9.0 %)	92 (9.9 %)	45 (3.1 %)	828 (100 %)
Both aripiprazole and risperidone in use	45 (0.49 %)	5 (0.53 %)	45 (3.1 %)	45 (5.4 %)
Clozapine in use	2249 (24.4 %)	159 (17.1 %)	403 (28.2 %)	72 (8.7 %)
CYP2D6 phenotype: poor	820 (8.9 %)	81 (8.7 %)	160 (11.2 %)	62 (7.5 %)
CYP2D6 phenotype: intermediate	2503 (27.1 %)	259 (27.8 %)	363 (25.4 %)	235 (28.4 %)
CYP2D6 phenotype: normal	5357 (58.0 %)	528 (56.7 %)	820 (57.4 %)	484 (58.5 %)
CYP2D6 phenotype: ultrarapid	552 (6.0 %)	63 (6.8 %)	86 (6.0 %)	47 (5.7 %)

a Adverse effect information available from 8930 patients and 912 in the ANT-subgroup,

b Smoking information available from 8720 patients.

**Table 2.** DDD (defined daily dose) ratios of aripiprazole and risperidone^a^ in different CYP2D6 phenotype groups in all patients

	All CYP2D6 phenotype groups ± SD and *n*	Ultrarapid mean ± SD and *n*	Normal mean ± SD and *n*	Intermediate mean ± SD and *n*	Poor mean ± SD and *n*
Patients using aripiprazole^b^	1.28 ± 0.56 *n* = 1463	1.32 ± 0.58 *n* = 86	1.27 ± 0.56 *n* = 836	1.29 ± 0.55 *n* = 335	1.25 ± 0.58 *n* = 47
Patients using risperidone^c^	0.63 ± 0.39 *n* = 866	0.60 ± 0.35 *n* = 48	0.64 ± 0.40 *n* = 477	0.64 ± 0.35 *n* = 214	0.61 ± 0.36 *n* = 21

a DDD of aripiprazole is 15 mg, DDD of risperidone is 5 mg (Leucht *et al*., 2013).

b *p* = 0.83 between groups (ANOVA), *p* = 0.43 between ultrarapid vs other phenotypes (Mann-Whitney U).

c *p* = 0.91 between groups (ANOVA).

### Medication adherence

Table 3 displays the results of a logistic regression analysis used to examine factors associated with self-reported medication adherence. CYP2D6 phenotypes were associated neither in all patients nor in aripiprazole or risperidone users with self-reported medication adherence. Older age and greater number of psychotropic medications were associated with better adherence in both aripiprazole and risperidone groups. Living in supported housing was associated with better adherence among aripiprazole users (*p* = 0.033) and showed a borderline significant association among risperidone users *(p* = 0.052).

**Table 3.** Stratified logistic regression models of medication adherence among patients using aripiprazole or risperidone

	Aripiprazole users		Risperidone users	
	OR [95% CI]	p	OR [95% CI]	p
BMI	1.00 [0.99, 1.02]	0.68	1.00 [0.97, 1.04]	0.79
Age	**1.02 [1.00, 1.03]**	**0.005**	**1.01 [1.00, 1.03]**	**0.033**
Diagnosis: non-affective psychosis	1.19 [0.87, 1.62]	0.28	**1.59 [1.01, 2.48]**	**0.042**
Gender male	0.82 [0.61, 1.10]	0.18	0.72 [0.48, 1.08]	0.12
Number of psychotropic medications	**1.22 [1.09, 1.37]**	**<0.001**	**1.22 [1.04, 1.44]**	**0.014**
Current smoking	0.86 [0.64, 1.15]	0.30	0.90 [0.60, 1.35]	0.61
Living in supported housing	**1.62 [1.05, 2.56]**	**0.033**	1.73 [1.01, 3.08]	0.052
CYP2D6 Poor^a^	0.91 [0.58, 1.47]	0.70	0.77 [0.39, 1.64]	0.48
CYP2D6 Intermediate^a^	1.26 [0.89, 1.81]	0.20	1.05 [0.68, 1.63]	0.84
CYP2D6 Ultrarapid^a^	1.36 [0.74, 2.73]	0.35	1.80 [0.73, 5.45]	0.24

Note: In this model, an OR greater than 1 indicates better medication adherence.

a Normal CYP2D6 phenotype was used as the reference.

### Medication adverse effects

Table 4 presents the results of the logistic regression model used to evaluate medication adverse effects. Aripiprazole users with ultrarapid CYP2D6 phenotype had more medication adverse effects (*p* = 0.041). In both the aripiprazole and risperidone groups, a higher number of psychotropic medications was associated with increased adverse effects. Additionally, among risperidone users, younger age was significantly associated with more adverse effects (*p*<0.001).

**Table 4.** Stratified logistic regression models of medication adverse effect among patients using aripiprazole or risperidone

	Aripiprazole users		Risperidone users	
	OR [95% CI]	p	OR [95% CI]	p
BMI	1.00 [0.99, 1.02]	0.54	1.01 [0.99, 1.04]	0.29
Age	0.99 [0.99, 1.00]	0.17	**0.98 [0.97, 0.99]**	**<0.001**
Diagnosis: non-affective psychosis	0.99 [0.77, 1.26]	0.91	0.97 [0.67, 1.40]	0.85
Gender male	0.93 [0.74, 1.18]	0.56	0.80 [0.58, 1.09]	0.16
Number of psychotropic medications	**1.19 [1.09, 1.30]**	**<0.001**	**1.30 [1.15, 1.47]**	**<0.001**
Current smoking	0.87 [0.69, 1.10]	0.26	0.89 [0.65, 1.23]	0.49
Living in supported housing	0.94 [0.69, 1.27]	0.68	0.72 [0.49, 1.05]	0.088
CYP2D6 Poor ^a^	1.06 [0.73, 1.54]	0.77	1.63 [0.90, 3.05]	0.11
CYP2D6 Intermediate ^a^	1.07 [0.82, 1.41]	0.61	1.06 [0.75, 1.49]	0.75
CYP2D6 Ultrarapid ^a^	**1.71 [1.03, 2.90]**	**0.041**	1.52 [0.78, 3.03]	0.22

Note: In this model, an OR greater than 1 indicates more adverse effects.

a Normal CYP2D6 phenotype was used as the reference.

### Attitudes towards neuroleptic treatment

The ANT attitude scores showed a negative correlation with reported medication adverse effects (r = −0.14, *p*<0.0001). The lowest attitude scores were in aripiprazole users with ultrarapid CYP2D6 phenotype and risperidone user with poor CYP2D6 phenotype (Table 5). Aripiprazole users with ultrarapid CYP2D6 phenotype (*n* = 11 with attitude scores available) had 17.0 % lower mean attitude scores implying more negative attitudes to medication compared to other CYP2D6 phenotypes combined (Mann-Whitney, *p* = 0.032). In the subgroup of patients for whom ANT-attitude data were available (*n* = 931), only one patient using risperidone belonged to the CYP2D6 poor phenotype group. In beta regression model explaining ANT attitude (Table 6) aripiprazole use with ultrarapid CYP2D6 phenotype was associated with lower ANT attitude scores compared to normal CYP2D6 phenotype (*p* = 0.023). Also, among aripiprazole users, male gender was associated with worse ANT attitudes (*p* = 0.035).

**Table 5.** Attitudes towards Neuroleptic Treatment-attitude in different cytochrome P450 2D6 phenotype groups

	Ultrarapid mean ± SD and *n*	Normal mean ± SD and *n*	Intermediate mean ± SD and *n*	Poor mean ± SD and *n*
All patients^a^ (*n* = 931)	68.0 ± 17.3 *n* = 69	70.6 ± 16.2 *n* = 575	68.0 ± 17.5 *n* = 261	65.8 ± 19.6 *n* = 26
Patients using aripiprazole^b^ (*n* = 147)	59.1 ± 20.1 *n* = 11	72.4 ± 16.1 *n* = 91	69.1 ± 17.9 *n* = 41	65.0 ± 7.4 *n* = 4
Patients using risperidone^c^ (*n* = 92)	68.5 ± 17.9 *n* = 4	70.1 ± 17.5 *n* = 64	67.7 ± 23.2 *n* = 23	58 *n* = 1

a *p* = 0.19 between groups (Kruskal-Wallis),

b *p* = 0.10 between groups (Kruskal-Wallis), *p* = 0.03 between ultrarapid vs other phenotypes (Mann-Whitney).

c *p* = 0.78 between groups (Kruskal-Wallis).

**Table 6.** Stratified beta regression models of Attitudes towards Neuroleptic Treatment-attitude among patients using aripiprazole or risperidone

	Aripiprazole users		Risperidone users	
	β [95% CI]	p	β [95% CI]	p
Age	0.005 [−0.004, 0.013]	0.27	−0.002 [−0.010, 0.006]	0.685
Gender male	**-0.25 [−0.48, −0.018]**	**0.035**	−0.14 [−0.41, 0.13]	0.306
Number of psychotropic medications	0.024 [−0.063, 0.11]	0.59	0.079 [−0.021, 0.18]	0.123
CYP2D6 Poor^a^	−0.23 [−0.71, 0.25]	0.35	−0.21 [−0.77, 0.35]	0.454
CYP2D6 Intermediate^a^	−0.082 [−0.34, 0.18]	0.53	−0.11 [−0.43, 0.21]	0.502
CYP2D6 Ultrarapid^a^	**-0.48 [−0.89, −0.065]**	**0.023**	−0.22 [−0.89, 0.45]	0.520

Note: In this model, a negative β indicates less favourable attitudes toward antipsychotic treatment.

a Normal CYP2D6 phenotype was used as the reference.

## Discussion

Several recent studies have demonstrated the importance of pharmacogenetic variability on medication metabolism and exposure. To our knowledge this is the first study assessing the association between CYP2D6 phenotype and self-reported adherence, perceived medication adverse effects, and medication-related attitudes among aripiprazole and risperidone users. We hypothesised that participants with a poor or ultrarapid CYP2D6 phenotype would have challenges related to medication tolerability and efficacy, potentially stemming from suboptimal serum levels of medication, poor treatment response, increased adverse effects, or a combination of these factors. Consequently, we expected these individuals to exhibit poorer medication adherence, experience increased adverse effects, and have more negative attitudes towards antipsychotic medications.

The main finding of this study was that, among aripiprazole users, the CYP2D6 ultrarapid phenotype was associated with increased adverse effects and less favourable attitudes toward antipsychotic medications, while no such associations were observed among risperidone users. However, these associations did not translate into the anticipated differences in medication adherence. The results of this study are inconsistent with previous research on risperidone, which showed an increased likelihood of switching from risperidone to another antipsychotic among both ultrarapid and poor metabolizers (Jukic *et al*., 2019).

As the study was cross-sectional, we considered the possibility that risperidone may have already been switched to another antipsychotic medication due to adverse effects or lack of efficacy among ultrarapid and poor CYP2D6 metabolizers. However, there was no difference in the proportions of CYP2D6 ultrarapid and poor metabolizers between the risperidone or aripiprazole groups and those using other antipsychotics, suggesting that no such bias was present.

Aripiprazole users with an ultrarapid CYP2D6 phenotype displayed more adverse effects which could explain the observed worse attitudes towards antipsychotic treatment. Rapid fluctuations in aripiprazole concentrations among these ultrarapid metabolizers could contribute to increased adverse effects. Another possible explanation for the observed worse attitudes is that reduced serum levels in this subgroup could lead to a diminished treatment response, leaving patients less satisfied with their medication.

In the logistic regression model, older patients and those taking a greater number of psychotropic medications demonstrated better adherence to their medication. Among aripiprazole users, also living in supported housing was associated with better adherence. Older age typically correlates with improved adherence due to factors like maturity, more stable routines, better insight into their condition, and more established relationships with healthcare providers (Kuroda *et al*., 2008; Blaschke *et al*., 2012; Yeisen *et al*., 2017). A greater number of psychotropic medications could indicate more severe symptoms or higher relapse risks, motivating them to better adherence. Contrary to our findings, poorer adherence has also been associated with polypharmacy (Smith *et al*., 2020). It is notable that polypharmacy was common in our sample, and approximately one fourth of the patients were prescribed clozapine, which may have contributed to better adherence (Takeuchi *et al*., 2020; Brodeur *et al*., 2022). Supported housing can enhance medication adherence for individuals with psychosis by providing a structured and stable environment, professional support, crisis management, and integrated care, thereby preventing disruptions in medication adherence (Leff, 2000; Poppe *et al*., 2024).

### Strengths and limitations

The key strengths of this study include a large national cohort of patients at various stages of psychosis and the availability of longitudinal national registry data. The ANT scale provides a more reliable and detailed measure of medication adherence compared to verbal inquiry, with medication-related attitudes strongly linked to adherence. Furthermore, non-adherence is associated with poorer treatment response. The ANT scale demonstrates good test-retest reliability and validity and enables quick and easy rating. However, its VAS structure may lead to high variance in single items between test situations (Kampman *et al*., 2000).

Certain limitations should be acknowledged. This study relies on self-reported information, which may introduce bias or inaccuracies, potentially impacting the reliability of the findings. The ANT-scale was available only for a subgroup of 1,000 patients from one region in Finland. For the attitude-related analyses, the number of covariates in the beta regression model was reduced, which limits our ability to fully account for potential confounding effects. The groups of aripiprazole and risperidone users with ultrarapid or poor CYP2D6 phenotypes were small in the ANT-scale subgroup, requiring cautious interpretation of our results. This study was cross-sectional, and we did not have data on how many antipsychotic medications were used in the past or on how long the current or previous antipsychotics were taken. These factors could potentially influence the outcomes. Furthermore, we had no baseline measures for the questionnaires used, so we were unable to compare changes in perceptions since starting treatment. Psychosis diagnoses for 122 patients (1.2%) could not be confirmed from registries and were instead based on self-report (Ahti *et al*., 2022). Private outpatient services are not covered by the registers, and the sample consisted of more long-term than acute patients, without data on patients’ current psychopathology.

In the Finnish population, the prevalence of the CYP2D6 UM phenotype is approximately 7%, significantly higher than the 2% observed in other Northern European countries. Conversely, the prevalence of the CYP2D6 PM phenotype is negligible in Finland, compared to around 7% in other Northern European populations. (Sistonen *et al*., 2007; Pietarinen *et al*., 2016; Häkkinen *et al*., 2022) The high frequency of CYP2D6 UMs in Finland enhances this study by providing sufficient statistical power to detect associations that might be overlooked in populations with lower UM prevalence. However, this same characteristic limits the study’s ability to identify significant effects related to the PM phenotype, due to its negligible occurrence in the Finnish population.

The DDD method was used to define drug doses, consistent with numerous prior studies. However, it is important to recognise that the DDD method is developed for drug consumption research, not for defining equivalent doses between different medications. Older antipsychotics may have relatively higher DDDs than newer drugs. DDD values typically remain unchanged despite new efficacy information in clinical practice to maintain consistency for long-term studies on drug consumption. (Leucht *et al*., 2016; WHO, 2022) Serum concentrations of medication would offer more accurate predictions of medication usage, but this study did not have access to serum concentration data.

## Conclusions

Previous studies suggest that pharmacogenetic testing could be beneficial in guiding dosing decisions for aripiprazole and risperidone and when there are multiple antipsychotics in use, particularly when patients experience adverse effects or when the current pharmacotherapy proves ineffective. In line with suggestions for pharmacogenetic testing, our findings indicate that the CYP2D6 ultrarapid phenotype was associated with increased adverse effects and poorer attitudes toward medications in aripiprazole users, whereas this association was not observed among risperidone users. Although the associations reached statistical significance, their proximity to the threshold suggests a borderline effect, and the small sample size of the ANT subgroup warrants cautious interpretation. Moreover, the potential impact of poor metabolizer phenotypes may have been underestimated due to their low prevalence in the Finnish population. Further research is needed to deepen the understanding of the impact of CYP2D6 phenotype on the tolerability and efficacy of aripiprazole and risperidone and to support the development of personalised medication approaches for these medications.
